# Cost-Utility Analysis of Lopinavir/Ritonavir versus Atazanavir + Ritonavir Administered as First-Line Therapy for the Treatment of HIV Infection in Italy: From Randomised Trial to Real World

**DOI:** 10.1371/journal.pone.0057777

**Published:** 2013-02-27

**Authors:** Emanuela Foglia, Paolo Bonfanti, Giuliano Rizzardini, Erminio Bonizzoni, Umberto Restelli, Elena Ricci, Emanuele Porazzi, Francesca Scolari, Davide Croce

**Affiliations:** 1 CREMS (Centre for Research on Health Economics, Social and Health Care Management), University Carlo Cattaneo - LIUC, Castellanza, Varese, Italy; 2 Department of Infectious and Tropical Diseases, A. Manzoni Hospital, Lecco, Italy; 3 First and Second Departments of Infectious Diseases, L. Sacco Hospital Authority, Milan, Italy; 4 Department of Occupational Health Clinica L. Devoto Labour, Section of Medical Statistics and Biometry G.A. Maccacaro, Faculty of Medicine and Surgery, University of Milan, Milan, Italy; 5 Faculty of Health Sciences, University of the Witwatersrand, Johannesburg, South Africa; Groningen Research Institute of Pharmacy, United States Of America

## Abstract

**Objective:**

To estimate the lifetime cost utility of two antiretroviral regimens (once-daily atazanavir plus ritonavir [ATV+r] versus twice-daily lopinavir/ritonavir [LPV/r]) in Italian human immunodeficiency virus (HIV)-infected patients naïve to treatment.

**Design:**

With this observational retrospective study we collected the clinical data of a cohort of HIV-infected patients receiving first-line treatment with LPV/r or ATV+r.

**Methodology:**

A Markov microsimulation model including direct costs and health outcomes of first- and second-line highly active retroviral therapy was developed from a third-party (Italian National Healthcare Service) payer’s perspective. Health and monetary outcomes associated with the long-term use of ATV+r and LPV/r regimens were evaluated on the basis of eight health states, incidence of diarrhoea and hyperbilirubinemia, AIDS events, opportunistic infections, coronary heart disease events and, for the first time in an economic evaluation, chronic kidney disease (CKD) events. In order to account for possible deviations between real-life data and randomised controlled trial results, a second control arm (ATV+r 2) was created with differential transition probabilities taken from the literature.

**Results:**

The average survival was 24.061 years for patients receiving LPV/r, 24.081 and 24.084 for those receiving ATV+r 1 and 2 respectively. The mean quality-adjusted life-years (QALYs) were higher for the patients receiving LPV/r than those receiving ATV+r (13.322 vs. 13.060 and 13.261 for ATV+r 1 and 2). The cost-utility values were 15,310.56 for LPV/r, 15,902.99 and 15,524.85 for ATV+r 1 and 2.

**Conclusions:**

Using real-life data, the model produced significantly different results compared with other studies. With the innovative addition of an evaluation of CKD events, the model showed a cost-utility value advantage for twice-daily LPV/r over once-daily ATV+r, thus providing evidence for its continued use in the treatment of HIV.

## Introduction

The generalizability of cost-effectiveness data gleaned from multinational studies is being increasingly called into question. Unless contextualised in the country of reference [1]–[3], such data will fail to capture salient differences in clinical practice, population characteristics, health care costs, treatment preferences, and cost-opportunity of resources [4]. This is of relevance for institutional decision makers, who are more likely to be interested in the prompt availability of context-specific data than heterogeneous data reported in an international study [5].

Another critical issue is the need to understand the impact of the effectiveness of treatments and choices in clinical practice as may be obtained from real-life data rather than from a randomized clinical trial (RCT), particularly as regards chronic diseases. For example, highly active antiretroviral therapy (HAART) in Italy is currently reimbursed by the National Health Service (NHS), without any threshold of utilisation (as for new drugs). However, human immunodeficiency virus (HIV) infection, once a fatal condition and now considered a chronic disease, has driven up overall NHS expenditures, with the result that the Italian NHS, like other health systems, is facing a general scarcity of resources. The decisions taken by the London Consortium in the U.K. are another example of this problem [6].

The introduction of a new therapeutic intervention implies not only an evaluation of its effectiveness, but also its long-term economic impact on the overall health budget and expenditures, without which the feasibility of the decision to introduce it will remain elusive.

Numerous predictive models have been proposed to elucidate the dynamics and the possible long-term consequences of HIV infection in terms of costs and effectiveness. Previous studies have evaluated this problem using a cost-utility approach from the patients’ perspective and by studying their preferences in various life conditions. Furthermore, Simpson et al. [7]–[10] laid the basis for the development of a Markov model in HIV infection. This predictive model was used to analyze the results of the CASTLE study (an open-label international non-inferiority randomised study of the use of LPV/r vs. ATV+r in antiretroviral-naïve HIV-1-infected patients) [11], in terms of quality-adjusted life years (QALY) related to the patients’ health states and the associated costs. A later study [12] applied the Markov microsimulation model (based on the individual patient level) to HIV-infected patients in accordance with the most recent international guidelines on drug treatment and patients evaluation (e.g., using 8 instead of 12 health states), rate of opportunistic infections (OIs), AIDS diagnosis, coronary heart disease (CHD) events, and incidence of hyperbilirubinemia and diarrhoea.

In the present study, we added to the model long-term renal toxicity, i.e., chronic kidney disease (CKD), an important event associated with both HIV infection and HAART. To date, this variable has never been taken into account in the evaluation, although the EuroSIDA study had considered it in terms of incidence and associated factors [13].

Here, the model was used to estimate the real lifetime cost utility of two ARV treatment regimens (once-daily atazanavir + ritonavir [ATV+r] in combination with tenofovir-emtricitabine [TDF/FTC] versus twice-daily lopinavir/ritonavir [LPV/r]) in Italian HIV-infected patients naïve to ARV treatment

## Methods

**Figure 1:**
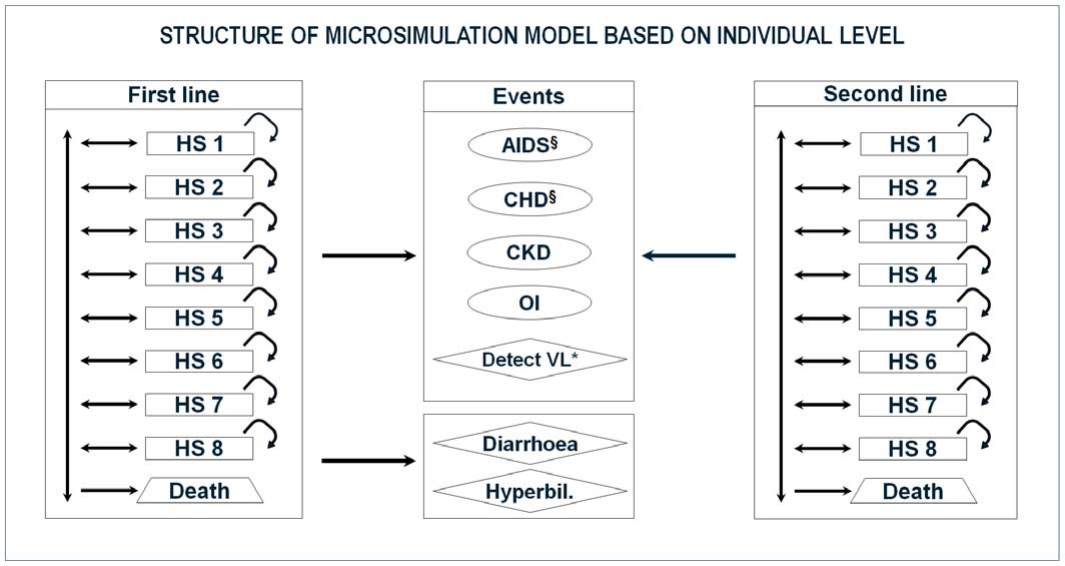
Structure of the microsimulation model at the individual level. Circle: event that does not determine a change of line of treatment. Rhombus: event that determine a change of line of treatment. HS: Health State. CHD: Coronary heart disease. CKD: Chronic kidney disease. OI: Opportunistic infection. VL: Viral load. § event that may lead to death. * Detectable viral load for two consecutive semesters. Patients enter the model being in first-line treatment (LPV/r or ATV+r). After each cycle, patients may change health state, die or experience events that may lead to a change in the line of treatment (patients in second-line had different treatment options that excluded those on first-line). Diarrhoea and hyperbilirubinemia may be experienced only by patients in first-line treatment, since these adverse events are associated with LPV/r and ATV+r therapies.

The Markov microsimulation model devised by Broder et al. [12] was further refined to create a new model (Fig. 1) from the NHS payer’s perspective that included direct costs and health outcomes of Italian HIV-infected patients receiving ATV+r or LPV/r.

### Population Sample

Patients attending the Infectious Disease Department 1, L. Sacco Hospital, Milan, were considered eligible to enter the study if diagnosed HIV-positive, were receiving first-line treatment with LPV/r or ATV+r, and had been followed up for 2 consecutive years after treatment initiation. Other inclusion criteria were: age >18 years old, HIV-1 RNA ≥5000 copies/mL, resident of Lombardy, and retrospectively identified from the Department clinical database of patients starting first-line treatment.

A consent form allowing the use of personal medical data was signed by the patients on their first visit to the Department to ensure anonymous data processing analysis. Base-case estimates are shown in Table 1.

**Table 1:** Base-case estimates used within the model

Parameter	Base-case estimate*	Reference
Male gender, %	75.9	Study database
Mean age ± SD	39.2 ± 9.5	Study database
Prior DM, %	3.8	Study database
Prior CHD, %	1.2	Study database
CHD risk, %		[17]
No prior DM/CHD	0.17	
Prior DM	0.82	
Prior CHD	3.75	
Prior DM/CHD	4.94	
Effect of treatment on TC:HDL ratio‡		[18]
LPV/r	–0.17	
ATV+r (1 & 2)	–0.40	
Risk of CHD being fatal, %	35.4	[19]
Effect of treatment on transition to HS with VL ≥50 copies/mL		[12]
LPV/r	Not Applicable	
ATV+r 1	Not Applicable	
ATV+r 2†	–19%	
AIDS risk (%)	0.09 to 94.35 (states 1 to 8)	Study database
CKD risk (%)		[13]
LPV/r	0.12 to 2.9 (cycle 1 to 40)	
ATV+r (1 & 2)	0.12 to 23.3 (cycle 1 to 40)	
OI risk (%)	1.76	Study database
Diarrhoea risk (%)		Study database
LPV/r	1.76	
ATV+r (1 & 2)	0.0	
Hyperbilirubinemia risk (%)		Study database
LPV/r	0.0	
ATV+r (1 & 2)	1.47	

SD: standard deviation; DM: diabetes mellitus; CHD: Coronary heart disease; TC: Total cholesterol; HDL: High-density lipoprotein; VL: Viral load; HS: Health state; OI: Opportunistic infection; CKD: Chronic kidney disease

* Estimates gathered from the literature and expressed as incidence rates (i.e., event/person-years) were converted into semestral probabilities using standard formulas

† Transition probabilities of transitioning to a state with greater viral load (≥ 50 copies/mL) are 19% lesser than LPV/r

‡ Effect on TC:HDL ratio is considered null in second-line treatment

Enrolment followed one of two scenarios: initiation of LPV/r or ATV+r (ATV+r 1), after which the patients remained on first-line treatment or switched to second-line treatment; the two lines were modelled at the individual patient level, in accordance with the analysis of the cohort study data.

### Model Characteristics

The patients on first-line therapy were categorized in eight health states according to CD4 cell count and viral load (HIV RNA copies/mL) (Table 2), as previously described [7], [8], [11], [12]. Patients with two consecutive detectable viral loads, or a primary treatment-related adverse event (diarrhoea or hyperbilirubinemia), or requiring a switch of drug regimen were assigned second-line treatment. The patients on second-line therapy had treatment options that excluded first-line options. Second-line treatment include all the treatment lines after the first line, and consider all the events and the HIV pathology evolution up to a patient’s death.

**Table 2:** Transition probabilities (per patient, per 6-month cycle) related to first-line treatment and second-line treatment and probabilities of AIDS event

Health state	CD4 (cell count /mL)	HIV RNA (copies/mL)	Transition ProbabilitiesLPV/r (first line)†	Transition Probabilities ATV+r 1 (first line)†	Transition Probabilities ATV+r 2* (first line)†	Transition Probabilities (second line)†	AIDS probabilities
1	> 350	< 50	0.70721	0.77487	0.7232	0.49504	0.0009
2	> 350	≥ 50	0.86111	0.71106	0.86980	0.60277	0.0162
3	201–350	< 50	0.38737	0.29664	0.40847	0.27118	0.0104
4	201–350	≥ 50	0.45253	0.36481	0.47386	0.31675	0.0585
5	50–200	< 50	0.05202	0.08941	0.05719	0.0364	0.0212
6	50–200	≥ 50	0.13132	0.09041	0.14281	0.09191	0.2276
7	< 50	< 50	0.01623	0.00485	0.01791	0.01134	0.6934
8	< 50	≥ 50	0.02844	0.02175	0.03135	0.01988	0.9435

† diagonal elements of transition matrix expressing the probability of remaining in the same health state after one Markov cycle (semester)

* transitions based on a 19% lesser likelihood of transitioning to a state with greater viral load than when receiving LPV/r

Transition probabilities for first-line health states (Table 2) were estimated from a sample of ARV-naïve patients starting HAART with LPV/r or ATV+r [14]. Second-line transition probabilities were estimated from the same database, but considering all second-line patients except those on LPV/r or ATV+r.

The patients’ progression through the health states was evaluated within a time frame of 6 months, in accordance with Italian clinical guidelines [15]. Health and monetary outcomes, associated with the long-term use of ATV+r and LPV/r regimens, were estimated for the eight health states, the incidence of diarrhoea and hyperbilirubinemia, AIDS events, opportunistic infections (OIs), coronary heart disease (CHD), and chronic kidney disease (CKD). The total cost per patient was calculated considering drug costs and patient’s clinical conditions (HS), as well as institutional guidelines, protocols and the reimbursement rates applied by the Region of Lombardy. Cost data and QALYs were discounted at an annual rate of 3% [16].

Based on the probabilities analysed in the reprocessing of the database, the microsimulation model was populated with a cohort of 500,000 subjects in each arm. In order to determine whether there was a significant deviation between real-life data and RCT results, a second control arm (ATV+r 2) was introduced, whereby the differential effectiveness results reported in the CASTLE study were applied to the transition probabilities of ATV+r 2.

The aim of these scenarios, i.e., the real-life situation and the scenario adjusted considering the results of the CASTLE study, was to forecast the lifetime development of the population.

The economic model was built and analysed using TreeAge Pro Suite 2010 (TreeAge Software Inc., Williamstown, MA).

### Event Rates

The model included different types of events: AIDS, CHD, OIs, diarrhoea, hyperbilirubinemia, and CKD. The AIDS event rate was related to the health state of each patient, according to CD4 cell count and viral load, whereas CHD incidence was calculated as the pooled risk of low, medium and severe events (angina, ischemic diseases, heart failure, acute myocardial infarction and stroke). The CHD risk was related to diabetes, prior CHD events and to total cholesterol (TC)/high-density lipoprotein (HDL) ratio, wherein a unit decrement in the ratio predicted a 14% reduction in CHD risk [17].

As described by Broder [12], Simpson [8], and the CASTLE study [18], the cardiovascular risk was adjusted to take into account that few CHD events are fatal [19].

Since the timelines of the model output were 2, 5, 10, 20 years, and lifetime, it was not possible to use the Framingham algorithm due to the gap in time. Therefore, the same assumptions as Broder et al. 2011 had made [12] were adopted. In our model (Fig. 1), CHD and AIDS events could lead to death.

Within the sample, the OI rate included tuberculosis, oesophageal candidiasis and gastric candidiasis, and the rate of adverse events was calculated from their occurrence (any grade gastrointestinal adverse event and hyperbilirubinemia) in the sample during the follow-up period.

The incidence of CKD was based on the EuroSIDA study [13], which categorised kidney disease into five levels, from low (glomerular filtration rate [GFR] 89–60 ml/min) to severe (GFR <15 ml/min). Rates were calculated in CKD severity strata, using clinical guidelines [20], and their distribution in the HIV-infected population. CKD rates increased steadily every 6 months in the patients receiving first-line therapy, whereas the CKD rate for the second-line patients remained unchanged at the level observed in the last 6 months when they were on first line. OIs and CKD were not considered to affect mortality or treatment effectiveness.

### Costs

The economic resource categories were: antiretroviral (ARV) drugs; outpatient and specialist services; other medicines purchased locally; and hospital admissions. Unlike those for AIDS, CHD, OIs, AEs and CKD, the ARV costs were calculated separately for each treatment arm. Hospital admissions were associated with HIV infection but not attributable to AIDS, CHD, OIs, drug-related adverse events or CKD. The cost of ARV drugs was derived from the Official Lombardy Region Bulletin [21].

The cost for each of the eight health states was estimated from the actual resource consumption recorded in the Lombardy Region databank (Lombardy Region Integrated Patient Database). The data represent the actual consumption of NHS resources in a real-life situation. They were reprocessed considering the reference cohort.

The cost of an AIDS event was calculated on the basis of Diagnosis Related Group (DRG) reimbursement, paid by the Lombardy Region, and did not take into account the patient’s health state.

The cost of a CHD event was calculated on the basis of event severity and weighted with the respective incidence in the population and derived from an average of the DRG if hospitalisation, diagnostic procedures, specialist visits, and treatments were consumed.

The cost calculated for OIs depended on the event: for tuberculosis it was DRG reimbursement, drug treatment and diagnostic tests, whereas for oral candidiasis it was specialist visit, diagnostic tests and drug therapy. The pooled results were calculated according to the respective incidence rates. Diarrhoea and hyperbilirubinemia costs were derived from the costs for diagnostic tests and treatment, as indicated by clinical guidelines. Just as for CHD, the CKD cost was similarly calculated according to severity and weighted with respective incidence rates [13], [20]. Besides hospital admissions, diagnostics, specialist visits, and chronic drug treatments, the cost estimate included the likelihood of a more invasive and expensive therapy such as dialysis. Table 3 reports the costs of treatment and adverse events per health state. For all events, the same cost was considered for each health state, whereas the treatment costs differed between treatment arms and health states.

**Table 3:** Costs of treatments and adverse events in euro for each Health State.

	LPV/r	ATV+r 1 and 2
Health State		
1	€ 11,423.24	€ 11,439.40
2	€ 11,563.34	€ 11,579.48
3	€ 11,621.42	€ 11,637.56
4	€ 11,575.64	€ 11,591.78
5	€ 11,837.66	€ 11,853.80
6	€ 11,753.78	€ 11,769.94
7	€ 11,895.84	€ 11,912.00
8	€ 11,409.78	€ 11,425.94
AIDS event	€ 4,684.00	
Coronary Heart Disease	€ 1,354.07	
Opportunistic Infection	€ 2,110.69	
Hyperbilirubinemia	€ 27.61	
Diarrhoea	€ 50.82	
Chronic Kidney Disease	€ 1,185.36	

The second-line treatment costs were calculated using the same method. All other treatment options recommended by the Italian guidelines were compared with the rate of use observed in the reference cohort.

### Health-Related Quality of Life

The model utilities (Table 4) were not related to treatment regimens, but instead to the health state specific for progression of disease. Since QALY data for the Italian HIV-positive population are not currently available in the literature, published data on the U.S. HIV-infected population were used [7], categorizing the patients in eight health states, as suggested by Broder et al. [12].

**Table 4:** QALY variables related to HS, and events entered in the microsimulation model.

HS	QALY applied to HS without CHD [9], [12]	QALY applied to HS with CHD [9], [12]	QALY AIDS [28]	QALY OI [29]	QALY Hyperbilirubinemia [23]	QALY Diarrhoea [23]	QALY CKD [13], [30]
1	0.9440	0.6006	0.5600	0.6200	0.8835	0.8273	0.8835
2	0.9350	0.6000	0.5600	0.6200	0.8824	0.8257	0.8824
3	0.9290	0.5996	0.5600	0.6200	0.8816	0.8245	0.8816
4	0.9320	0.5998	0.5600	0.6200	0.8820	0.8251	0.8820
5	0.8630	0.6002	0.5600	0.6200	0.8725	0.8111	0.8725
6	0.8490	0.6007	0.5600	0.6200	0.8704	0.8080	0.8704
7	0.7810	0.6005	0.5600	0.6200	0.8592	0.7913	0.8592
8	0.7810	0.6005	0.5600	0.6200	0.8592	0.7913	0.8592

HS: Health state; QALY: Quality-adjusted life years; CHD: Coronary heart disease; CKD: Chronic kidney disease; OI Opportunistic infection

In our model, CHD events led to a 40% decrease in the quality of life (QoL), as reported in Castiel et al. [22], whereas in the microsimulation model, the QoL worsened progressively from health state 1 to 8; in the second-line treatment the same QALY of health states 7 and 8 was assigned to each health state.

Diarrhoea and hyperbilirubinemia utility values associated with events were selected by analysing the literature [23]–[25] and by applying the Delphi technique [26]–[27]. This involved ten infectious disease specialists working in the Lombardy Regional Healthcare Service. A questionnaire, starting with open questions and ending with closed questions, was successively administered to the experts to determine, starting from the utility values reported in the literature, which value should be associated to each event. The same methodology was used to determine the utilities due to AIDS events [28], OIs [29], and CKD [30].

### Mortality

The mortality rate of the HIV-infected population was derived from the mortality rate reported for the general Italian population [31], stratified by age and gender, and adjusted for major risk associated with HIV seropositivity, using the mortality rate ratio (MRR) reported for the European HIV-infected population [32].

### Sensitivity Analysis

A probabilistic sensitivity analysis was performed for both scenarios (LPV/r vs. ATV+r 1 and LPV/r vs. ATV+r 2) by varying the probability values of a CHD event being fatal; CKD, OIs, diarrhoea and hyperbilirubinemia rates; effect of treatments on TC:HDL ratio (ranges to cover up to 10-fold change from base case, as reported in Broder et al., 2011 [12]); utility values associated with health state, CHD events, diarrhoea, hyperbilirubinemia, CKD, AIDS events, and OIs; cost values of health state, CHD events, CKD, OIs; and the transition probabilities for ATV+r 2 (Table 5).

**Table 5:** Parameters used within the sensitivity analysis performed.

Parameter	Range for sensitivity analysis*	Reference
Risk of CHD event being fatal	Uniform (0.254, 0.454)	[12]
Diarrhoea risk‡	Beta (8, 448)	Study database
Hyperbilirubinemia risk‡	Beta (3, 189)	Study database
CKD risk	Uniform (± 10% of base-case value)	Expert opinion
OIs risk‡	Beta (11, 637)	Study database
Effect of treatment on TC:HDL ratio		[12]
LPV/r	Normal (–0.17, 1.56)	
ATV+r (1 & 2)	Normal (–0.40, 2.82)	
Effect of treatment on transition to HS with VL ≥ 50 copies/mL		Expert opinion
LPV/r / ATV+r 1	Not Applicable	
ATV+r 2	Uniform (–0.29, –0.09)	
HS QALY weight	Uniform (± 5% of base-case value)	Expert opinion
CHD QALY weight	Uniform (± 10% of base-case value)	Expert opinion
Diarrhoea QALY weight	Uniform (± 10% of base-case value)	Expert opinion
Health state QALY weight	Uniform (± 10% of base-case value)	Expert opinion
Hyperbilirubinemia QALY weight	Uniform (± 10% of base-case value)	Expert opinion
CKD QALY weight	Uniform (± 10% of base-case value)	Expert opinion
AIDS QALY weight	Uniform (± 10% of base-case value)	Expert opinion
OI QALY weight	Uniform (± 10% of base-case value)	Expert opinion
HS cost (first and second line)	Uniform (± 3% of base-case value)	Study database
CHD cost	Uniform (± 20% of base-case value)	Study database
CKD cost	Uniform (± 5% of base-case value)	Study database
OI cost	Uniform (± 10% of base-case value)	Study database

CHD: Coronary heart disease; CKD: Chronic kidney disease; OI: Opportunistic infection; TC: Total cholesterol; HDL: High-density lipoprotein; HS: Health state; QALY: Quality-adjusted life years.

* Ranges are: minimum and maximum or percentage variation of base-case values for uniform distributions; mean and standard deviation for normal distributions; alpha and beta are shape parameters for beta distributions.

‡ Risk values of diarrhoea, hyperbilirubinemia and opportunistic infections distributed according to a beta probability distribution.

A total of 200 microsimulations were generated for each analysis, each populated with 5,000 individuals.

## Results

In this section the results of the two analyses are presented separately, both LPV/r vs. ATV+r 1, based on real life data, and LPV/r vs. ATV+r 2, where the efficacy of ATV+r 2 arm is calculated as a differential value, as reported in the CASTLE study.

### LPV/r vs. ATV+r 1

QALY values were calculated by reprocessing the data obtained from several sources, as described in the Methods section. Whilst AIDS and OI QALY did not vary across health states, QALY values steadily decreased across the health states for CHD, AEs, and CKD (Table 4). When these results were included in the model, the scenarios showed an average survival, in years, of 24.061 for the LPV/r arm and 24.081 for ATV+r 1 (Table 6).

**Table 6:** Lifetime results of the model divided per treatment.

Parameter	LPV/r	ATV+r 1	ATV+r 2
Survival years (mean)	24.061	24.081	24.084
QALYs (mean)	13.322	13.060	13.261
Per capita mean annual cost (€)	8,477.09	8,624.77	8,548.21
Total cost (€)	203,967,086	207,693,086	205,875,090
Total cost per QALY (€)	15,310.56	15,902.99	15,524.85
Years on first-line therapy (mean)	11.711	11.143	13.466
Patients ending in first-line therapy (%)	33.43	30.92	41.12
Patients with at least 1 CHD event (%)	9.94	9.81	9.77
Patients with at least 1 CKD event (%)	41.34	58.93	63.65
Total CHD events (events per 1000 patient-years)	6.6	6.5	6.4
Total AIDS events (events per 1000 patient-years)	49.5	54.9	46.1
Total CKD events (events per 1000 patient-years)	27.1	110.9	135.3
Total OI events (events per 1000 patient-years)	35.4	35.4	35.4
Total treatment emergent AE (events per 1000 patient-years)	17.7	14.1	16.9
Two consecutive cycles with VL ≥ 50 copies/mL (events per 1000 patient-years)	17.2	20.4	14.3
Total patient-years	12,030,423	12,040,386	12,041,769
Population size	500,000	500,000	500,000

QALY: Quality-adjusted life years; CHD: Coronary heart disease; CKD: Chronic kidney disease; OI: Opportunistic infection; AE: Adverse event; VL: Viral load.

The mean QALYs were higher in the LPV/r arm than in the ATV+r 1 (13.322 vs. 13.060), with a gain of 0.262.

The results showed a similar incidence of cardiovascular events in the LPV/r and ATV+1 arms (6.6 cases per 1,000 patient years vs. 6.5 per 1,000 patient years). The incidence of CKD was lower in the LPV/r arm than in the ATV+r 1 arm (27.1 cases vs. 110.9 per 1,000 patient years).

There was a higher incidence of drug-related adverse events among the patients receiving LPV/r than among those in the ATV+r 1 arm (17.7 vs. around 14.1 per 1,000 patient years).

Although the use of LPV/r showed an advantage in terms of costs, the economic weight of kidney-related events had a significant impact on the final results, leading to an advantage for the use of LPV/r, with a lower rate of CKD events among the patients receiving LPV/r than among those in the ATV+r 1 arm. However, a higher rate of CHD events was observed in the patients receiving LPV/r than in those in the ATV+r 1 arm.

The annual cost analysis showed nearly complete overlapping of the economic performance of the two treatments: the LPV/r arm had a per capita annual advantage of €148 in comparison with the ATV+r 1 arm. In addition, the cost-utility values (€15,310.56 for LPV/r; €15,902.99 for ATV+r 1) confirmed an advantage in the use of the LPV/r regimen.

**Figure 2:**
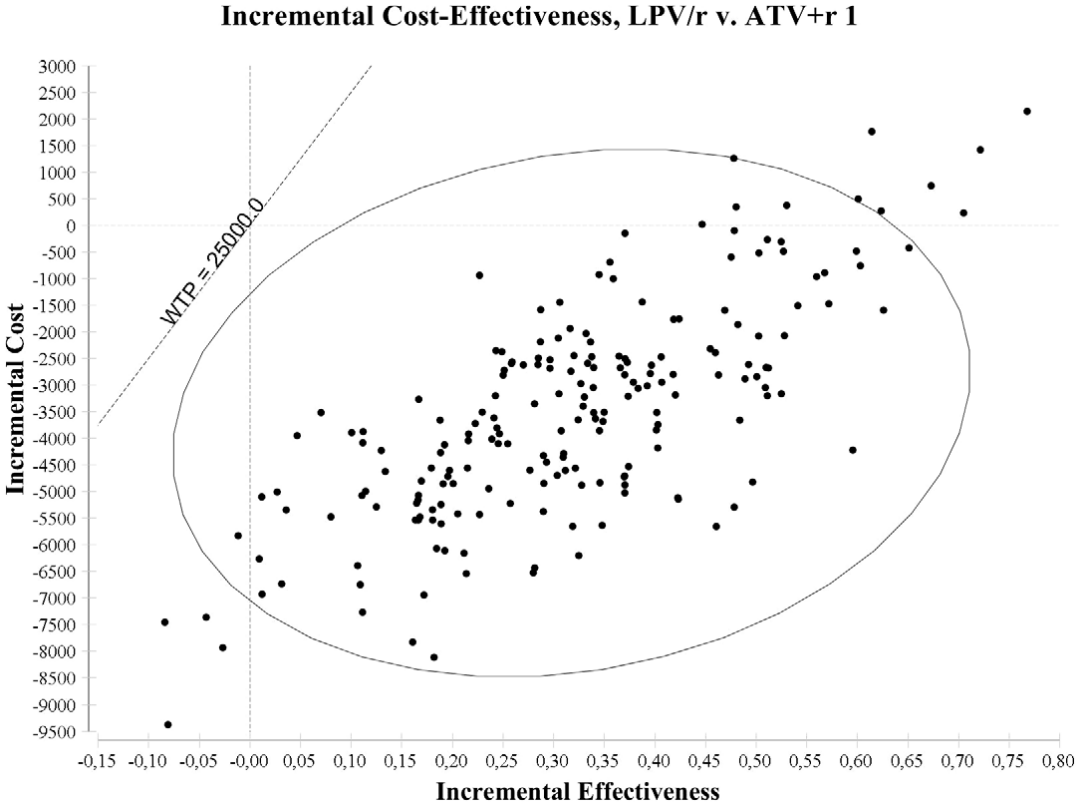
Incremental cost effectiveness ratio plan, presenting the results of the probabilistic sensitivity analysis of LPV/r vs. ATV+r 1 regimens.

Figure 2 reports the results of the probabilistic sensitivity analysis for LPV/r vs. ATV+r 1. Considering a €25,000 willingness to pay threshold [33], the LPV/r regimen had a lower cost and higher effectiveness, being the dominant alternative, in 92.0% of the simulations, higher effectiveness and cost with an incremental cost-effectiveness ratio (ICER) <25,000 in 5.5% of the simulations, and lower effectiveness and cost with an ICER >25,000 in 2.5% of the simulations, being cost effective at a €25,000 per QALY willingness to pay in 97.5% of the simulations.

### LPV/r vs. ATV+r 2

The average survival of patients receiving ATV+r 2 was 24.084 years (Table 6), almost identical to that noted for the LPV/r arm. The mean QALYs were slightly higher in LPV/r arm than in the ATV+r 2 arm, with a 0.061 gain (13.322 vs. 13.261).

A marginally higher incidence of cardiovascular events was associated with LPV/r in comparison with ATV+r 2 (6.6 vs. 6.4 cases per 1,000 patient years), with a lower incidence of CKD (27.1 vs. 135.3 cases per 1,000 patient years), and a higher incidence of drug-related adverse events (17.7 vs. 16.9 per 1,000 patient years).

The ATV+r 2 arm was more cost advantageous in the shorter time period, i.e., projections of up to 20 years, while LPV/r showed an advantage in the lifetime analysis. The annual cost showed a slight advantage for LPV/r, with a cost-utility value of €15,310.56 for LPV/r and €15,524.85 for ATV+r 2, respectively.

**Figure 3:**
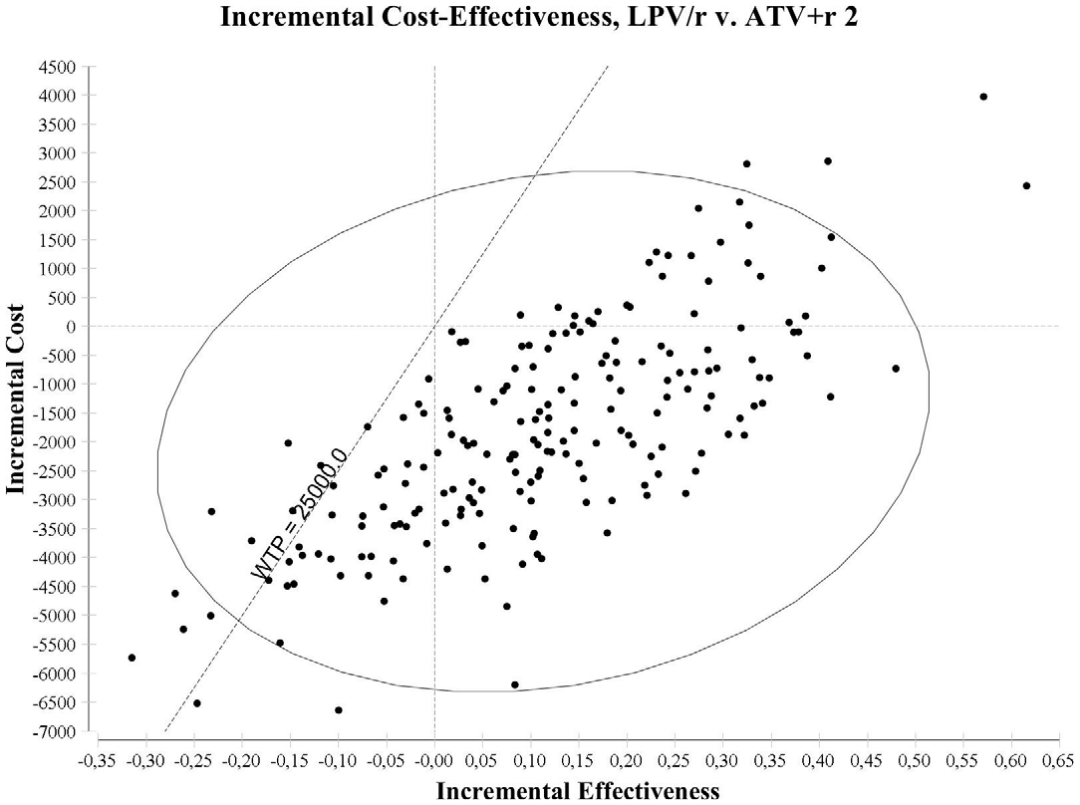
Incremental cost effectiveness ratio plan, presenting the results of the probabilistic sensitivity analysis of LPV/r vs. ATV+r 2 regimens.

Figure 3 reports the results of the sensitivity analysis of LPV/r vs. ATV+r 2. LPV/r had lower costs and higher effectiveness, being the dominant alternative, in 61.0% of the simulations, higher effectiveness and costs with an ICER <€25,000 in 15.0% of the simulations, lower effectiveness and cost with an ICER <€25,000 in 5.0% of the simulations, and lower effectiveness and higher cost with an ICER >€25,000 in 19.0% of the simulations, being cost effective at €25,000 per QALY willingness to pay in 81.0% of the simulations.

## Discussion

In both analyses (LPV/r vs. ATV+r 1 and LPV/r vs. ATV+r 2), LPV/r is dominant compared to ATV+r, resulting in a lower lifetime cost and in a higher utility value.

This Markov model was based on regional and national data. Its potential is a closer alignment of the results to specific regional or country-based protocols and costs, with greater usability from a payer's perspective for decision-making processes. The clinical results are consistent with previous data on ATV and LPV [10]–[12]. In addition, the new information gained by including CKD in the computations builds on current cost-utility analyses in this field.

Since the objective of this analysis was to provide Italian policy makers with information cogent to occurring events of the surveyed area, the cost data are based on a specific national analysis rather than on international assessments that do not take into account the clinical practice protocols currently in use. As such, it gives a fairly accurate picture of what is now happening in Lombardy (Italy’s most populous region, with more than 15% of the total population) [34].

Although the sample is restricted to Lombardy, the guidelines for the management of HIV-infected patients are national. Furthermore, the Italian NHS is based on a regional reimbursement system, with no significant differences among the country’s regions; therefore, the sensitivity analysis could cover these differences, providing useful information to policy makers in any region.

Using a patient-based methodology it is possible to follow the diagnostic and care pathway of patients and understand their actual consumption of resources. This makes the results of the cost analysis even more relevant, moreover, unlike a theoretical value, cost data were affected by the compliance of patients.

The main limitation of the study is the low number of patients enrolled in the ATV+r arm, primarily because ATV+r became available after LPV/r. This limitation was overcome by using a second ATV+r arm that took into account (as in Broder et al. [12]) a lesser probability of transitioning to a state with a greater viral load than the LPV/r arm, therefore more consistent with the CASTLE trial results and providing the possibility to verify the robustness of the results. A second limitation of the study was that some assumptions cannot be based on Italian data. For example, there is a lack of information about QALYs in the Italian HIV-infected population.

The MRRs were calculated by adjusting the mortality data for the general Italian population for major risks linked to HIV infection, and derived from a European HIV-infected population, as reported by Obel et al. [32]. Their study was selected because the distribution of risk factors was similar to our study sample for gender, risk factors for HIV acquisition, and hepatitis C virus co-infection. The MRRs were not the actual MRRs of the Italian HIV-infected positive population, however.

The model was innovative in that it included CKD events, a variable not considered in previous studies for a cost-utility analysis. Such events, due to their high probability to occur, can significantly influence the results, when compared with similar published studies on the economic impact of the two treatment regimens. The results of the study presented, however, are consistent with data from the EuroSIDA study [13]. Moreover, unlike previously published models, where second-line treatment was simulated at the cohort level by employing a deterministic methodology, our model extends the simulation at individual patient level to second-line treatment, thus enhancing the realism of the analysis.

CKD events had an impact on costs and on QALYs but not on mortality rate. As CKD is a chronic degenerative condition, it incurs more costs and diminishes the patient’s quality of life.

There is a growing body of literature on CKD and HAART in HIV infection. A 2012 study [6] reviewed the literature on the incidence of CKD, in particular in patients receiving ATV. In order to analyse what impact this would have on the model, specific Italian-based studies will need to be implemented to single out the side effects of tenofovir and ATV.

In our sample, the distribution rate of AIDS, which reflects the frequency of event occurrence, was in line with the principal literature.

Despite the substantial overlap of the results for the different populations studied and the assessment methods used, albeit more conservative and closer to real life, the combination of cost data with utilities data shows a dominance of LPV treatment compared with ATV. Hence, LPV treatment being less expensive and more effective, it was not necessary to calculate the ICER.

The results of the present study underline the importance of collecting real-life data, which may differ from those reported in a RCT, in order to support decision makers in the Italian NHS and other health services as they contend with dwindling resources.

Also, the study results demonstrate the importance of ensuring the clinical quality of inputs into a model where the use of theoretical assumptions, not tested in clinical practice, might produce unreliable data for clinicians.

Undoubtedly, RCTs furnish an important and irreplaceable source of information on treatment efficacy; nonetheless, observational studies based on regional or national real-life clinical and economic data, are also essential to evaluate the real impact of treatments (as underlined by agencies worldwide [35]) and to take decisions at the regulatory level.
