# Correction: Cost-Utility Analysis of Lopinavir/Ritonavir versus Atazanavir + Ritonavir Administered as First-Line Therapy for the Treatment of HIV Infection in Italy: From Randomised Trial to Real World

**DOI:** 10.1371/annotation/e57d7e73-826c-4e1c-a7c6-d2c32d3809af

**Published:** 2014-01-03

**Authors:** Emanuela Foglia, Paolo Bonfanti, Giuliano Rizzardini, Erminio Bonizzoni, Umberto Restelli, Elena Ricci, Emanuele Porazzi, Francesca Scolari, Davide Croce

There is an error in Table 6. The vales in line 5 "Total cost (€)" should be listed in thousands, not millions. Please view the correct table here: http://plosone.org/corrections/pone.0057777.t006.cn.tif

